# A genetically encoded reporter reveals interferon responses in multiple cell lineages

**DOI:** 10.64898/2026.01.08.698513

**Published:** 2026-01-10

**Authors:** Sarah R. Anderson, Pailin Chiaranunt, Nicholas M. Mroz, Sarah D. Wade, Ari B. Molofsky, Anna V. Molofsky

**Affiliations:** 1 Department of Psychiatry and Behavioral Sciences/Weill Institute for Neurosciences.; 2 Department of Laboratory Medicine, University of California, San Francisco, San Francisco, CA.; 3 Kavli Institute for Fundamental Neuroscience, University of California, San Francisco, San Francisco, CA.

## Abstract

Interferons (IFNs) are canonical antiviral cytokines with emerging homeostatic functions across tissues, including in the brain. To date, a lack of tools to visualize IFN-responsive cells has limited our understanding of the breadth and nature of IFN signaling. Here we developed a novel and bright mouse reporter of IFN responses called IFN-brite, Bright Reporter of Interferon sTimulated gene Expression, consisting of two copies of the bright and fast-maturing green fluorescent fluorophore mGreenLantern downstream of the native *Isg15* gene. *Isg15* is one of the most abundant interferon stimulated genes (ISGs) and is detected in many cell types, including immune, stromal, and neuronal cells. Using in vivo and in vitro cytokine delivery, we show that IFN-brite is preferentially sensitive to IFN-β, with detectable but lower sensitivity responses to IFN-γ. We also detect IFN-brite signals in most brain cell types and in stromal and hematopoietic cells in the lung after influenza A infection. These data define a broadly useful new tool for studying IFN responses and suggest that diverse cell types, including neurons, can respond to IFNs.

## Introduction

IFNs are an ancient yet highly divergent family of cytokines that co-evolved as an antiviral defense system (1, 2). Type I interferons are present in all vertebrates, although the cytokines themselves have undergone extensive gene duplication and diversification, resulting in multiple ligands, which include IFN-β, and >10 IFN-α genes, depending on species, and IFN-ε, IFN-κ, and IFN-ω. These IFN signal through the type I receptor heterodimer (IFNAR1/2), which are expressed in every cell in the body as an innate and immediate line of defense against viral pathogens (3). Type ll interferon signaling has a single ligand, IFNγ, and signals through a distinct receptor pair (IFNGR1/2) and is present only in mammals. Finally Type III, signalling via IFN-λ, evolved somewhat in parallel with the Type I family, but has some structural homology with IL-10 family cytokines, has a unique receptor, and may have more tissue specific functions (4), with receptors that are typically restricted to epithelial cells in barrier tissues and select immune cells(5). Despite these upstream differences, all three families of IFNs result in highly conserved JAK-STAT (Janus Kinase-Signal Transducer and Activator of Transcription) mediated transcriptional upregulation of interferon stimulated genes (ISGs) (6, 7). There are hundreds of ISGs with broad biological functions which together promote an efficient anti-viral response, but in addition, have increasingly appreciated physiologic roles in response to ‘vanishingly low quantities’ of cytokine (8). In particular, Type I interferon has homeostatic functions across tissues and cell types (7, 8) including in brain resident macrophages (microglia), where we identified a neuron-engulfing state that is required for healthy brain development (9).

Detecting and parsing cells that are IFN responsive has been challenging for several reasons. First, sensitivity of detection has been a limiting factor, particularly when studying tonic, or homeostatic IFN responses. Secondly, not all ISGs are induced in all cell types and contexts. Immunostaining for specific ISGs may detect cell types which employ that ISG for an IFN-mediated function that is not conserved in other cell types. Thirdly, because of the high conservation of signaling downstream of multiple IFN pathways, it is frequently not possible to distinguish Type I from Type II or III signaling. Other interferon reporters exist, such as one expressing green fluorescent protein (GFP) driven by the core transcription factor Mx1 (10), which can preferentially detect IFN-I responses(9). A separate reporter based on the ISG *Irgm1* responds to all three IFNs but is somewhat more sensitive to IFNγ (11). In both of the above cases, the sensitivity of these reporters to low level, tonic IFN signaling is limited. In addition, whether these tools can detect interferon responses in non-hematopoietic cells remains in question. After influenza infection, only hematopoietic cells expressed Mx1^GFP(10)^, whereas lung alveolar TypeI/II cells were detected with Irgm1^DsRed(11)^. Given the critical roles of IFN signaling across tissues, including in the central nervous system (CNS), we sought to develop a tool to examine IFN responses more broadly across cell types.

Here we describe a novel, highly sensitive reporter of IFN responses that can detect low levels of IFN signaling across multiple cell types, including in the brain. We used *in silico* analyses to identify an ISG that met the following criteria: 1) broadly expressed across all known cell types in response to IFN signaling, 2) robustly induced, and 3) not tonically expressed in an IFN-independent manner. We chose to use the endogenous promoter for *Isg15,* an ISG involved in “ISG-ylation” (a biochemical process similar to ubiquitination that modifies and regulates ISGs) (12), reasoning that a regulatory pathway would be more likely to be conserved across cell types. We generated a knock-in mouse model, IFN-brite, Bright Reporter of Interferon sTimulated gene Expression for *in vivo* and *in vitro* detection of IFN responses. We show that mice harboring this reporter are viable and healthy, and that the reporter can sensitively detect IFN responses in multiple cell types, including immune cells, stromal cells, and neurons throughout the body, including lung epithelium and brain. The reporter is highly sensitive to Type I signaling and with lesser affinity for Type II signaling. Our data suggest that this is a broadly useful tool to detect IFN-responses, particularly in physiology, and in cell types where they have been less studied, such as in neurons(13).

## Results

### Design and in vitro characterization of IFN reporter, IFN-brite.

**Figure 1:**
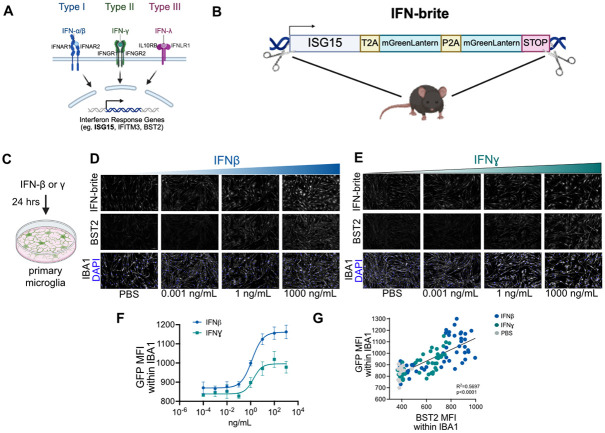
Design and in vitro characterization of IFN reporter, IFN-brite. A) Schematic of IFN signaling pathways and select ISGs, including *Isg15.* B) Design of IFN-brite reporter, which includes two copies of mGreen lantern inserted in the 3’UTR of the endogenous *Isg15* gene locus. C) Design of in vitro assay using mouse primary microglia to detect IFN responses. Indicated cytokines were added for 24 hours prior to fixation, antibody amplification, and imaging. D) Representative images of in vitro microglial responses to varying doses of IFN-β, showing expression of IFN-brite (anti-GFP antibody), and antibodies to the ISG BST2, and the marker gene IBA1. E) Representative images of in vitro microglial responses to varying doses of IFN-γ using the same imaging panel as in D. F) Best-fit curve of IFN-brite expression in microglia after 24 hours of varying doses of IFN-β and IFN-γ and by mean fluorescence intensity (MFI) of within masked IBA1 signal. n=6 wells, 2 independent experiments per group. G) Correlation of IFN-brite expression in microglia (by MFI) with protein staining of the ISG BST2 (a.k.a. tetherin).

*Isg15* is a canonical ISG expressed in multiple cell types and downstream of IFN-dependent JAK/STAT signaling (Fig. 1A). To generate the knockin mouse model IFN-brite (Bright Reporter of Interferon sTimulated genes), we used CRISPR-mediated recombination to insert a construct containing two copies(14) of mGreenLantern (mGL), a bright monomeric mutant GFP with a maturation half-time of 13.5 minutes(15). The construct was inserted just after the stop codon of *Isg15*, so that these would be expressed in-frame with native murine Isg15 gene product (Fig. 1B).

After validation of founders with correct insertion and lack of off-target mutations we performed *in vitro* assays to test the sensitivity and specificity to exogenous IFNs. We generated primary cultures of both microglia and astrocytes, and applied increasing concentrations of IFN ligands (IFNβ, IFNγ, IFNλ2). We first added IFNs to primary cultured microglia cells and performed fixation and imaging 24 hours later (Fig. 1C). We observed robust induction of IFN-brite signal within 24 hours of both IFNβ and to a lesser extent IFNγ application (Fig. 1D-E). While both reached their maximal response at a concentration of ~100 ng/mL, we observed about 50% stronger induction with IFNβ (Fig. 1F). This effect strongly correlated with protein levels of the ISG BST2(16) (Fig. 1G, R^2^=0.5697, p<0.0001). Shorter exposures (16 hours) were sufficient to induce IFN-brite in response to IFNβ but minimally in response to IFNγ (Fig. S1A-C). We did not detect any IFN-brite induction in response to IFNλ2 (Fig. S1D). As IFN-I and IFN-II responses are often co-occurring, these data cannot definitively distinguish whether IFN-brite is specific to either pathway, however, they suggest that it responds sooner and with a broader dynamic range to Type I vs. Type II IFN.

### In vivo characterization of IFN-brite in response to exogenous interferons.

**Figure 2:**
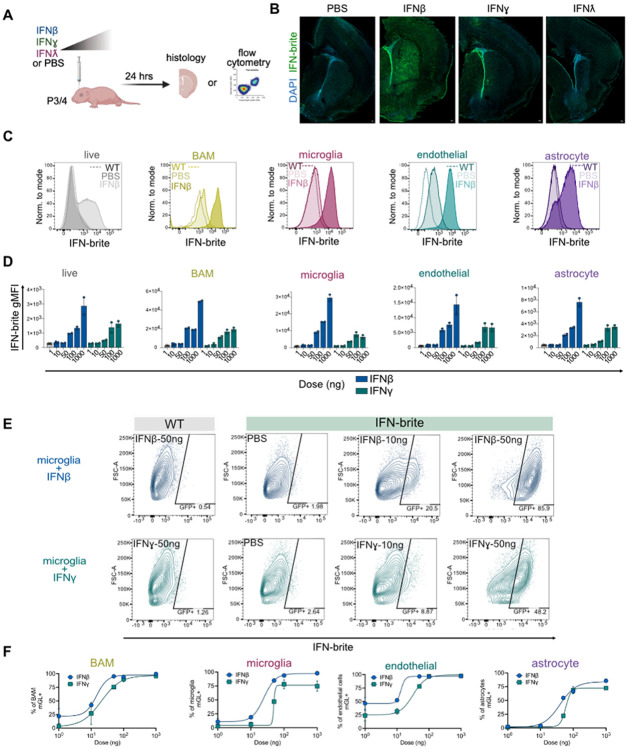
In vivo characterization of IFN-brite in response to exogenous IFNs. A) Schematic of in vivo dosing strategy to measure IFN-brite responses in brain cells B) Representative coronal sections of fixed and antibody stain mouse brain at P4-5 showing IFN-brite (GFP) and DAPI nuclear stain. Scale =100μm C) Representative histograms showing IFN-brite fluorescence by flow cytometry 24 hours after administration of 50ng of IFNβ per hemisphere dose in the indicated cell types. Plot shows WT (non-transgenic) animal with 50ng IFNβ, IFN-brite with vehicle administration (PBS), and IFN-brite with IFN-β. D) Quantification of IFN-brite geometric mean fluorescence intensity (gMFI) to varying doses of indicated cytokine. n=2-4 per group E) Representative contour plots of microglial response to varying doses of IFN-β showing gating strategy of IFN-brite positive cells. F) Representative contour plots of microglial response to varying doses of IFN-γ showing gating strategy of IFN-brite positive cells. G) Quantification shows best fit curve of % positive cells at indicated doses for both microglia, astrocytes, BAM, and endothelial cells in response to IFN-β or IFN-γ Abbreviations: BAM, border associated macrophages; WT, wild-type; ISG, interferon-stimulated gene.

To determine whether IFN-brite could report on IFN exposure *in vivo* within the CNS, we injected IFN ligands (IFNβ, IFNγ, IFNλ2) intracerebroventricularly (i.c.v.) at postnatal day 3-4 and collected brains 24 hours later for fixation and immunostaining (Fig. 2A; 50ng/0.5μl per hemisphere). IFNβ induced robust IFN-brite expression throughout the brain parenchyma, including cortex, striatum, and ependymal cells whereas IFNγ induced weaker and more localized responses around the ventricle and at the injection site at equivalent doses. IFNλ2 did not appreciably increase expression, consistent with our *in vitro* data (Fig. 2B).

We next examined and quantified CNS IFN responses by flow cytometry in microglia, border associated macrophages (BAM), endothelial cells, and astrocytes, using a cold protease digestion protocol to capture multiple cell types while limiting microglial activation(17) (gating strategy in Fig. S2A). We observed IFN-brite expression in all cell types, although the amount of basal expression and extent of induction varied by cell type (Fig. 2C, D). As with our *in vitro* data, we found a greater dynamic range of expression in response to IFNβ. At the highest dose tested (1000 ng), IFN-brite MFI was 2-4x stronger in response to IFNβ vs IFNγ.

We also quantified the percent of microglia and astrocytes detectably responding to IFNβ and IFNγ, gating against WT littermate controls. Although intensity of expression in response to IFNβ increased in a dose-dependent manner (Fig. 2C-D), the percent of microglia that had detectable IFN-brite expression reached saturation at ~100 ng per hemisphere (Figure 2E,F). We observed similar but more modest effects for IFNγ, and across other cell types, including astrocytes, endothelial cells, and BAMs (Fig. 2F). Together, this data suggests that IFN-brite can sensitively detect IFN responses across multiple cell types both by immunostaining and flow cytometry.

### IFN-brite detects brain IFN responses after cytokine exposure and during experience-dependent circuit remodeling.

**Figure 3:**
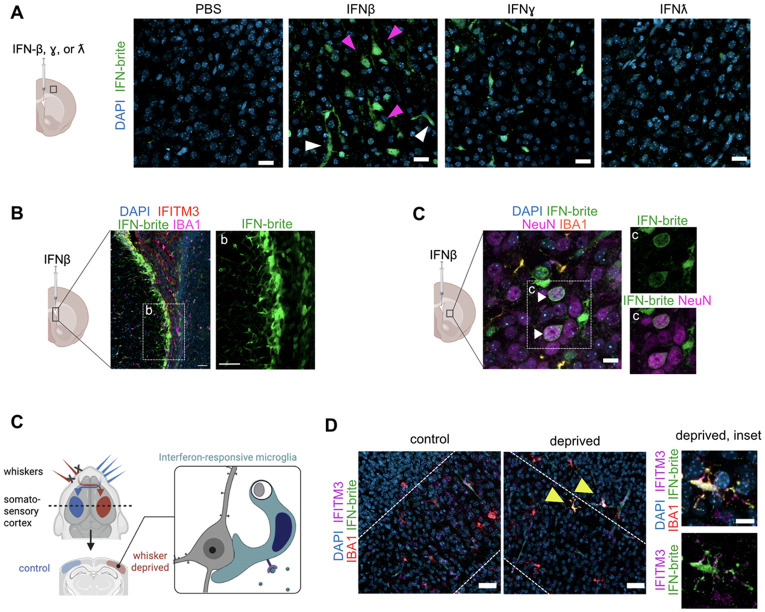
IFN-brite detects brain IFN responses after cytokine exposure and during experience-dependent circuit remodeling. A) Representative coronal sections of fixed and antibody stained layer 5 mouse somatosensory cortex (Layer 5) aged P4-5, 24 hours after intraventricular injection of indicated ligand showing IFN-brite (anti-GFP, green) and DAPI nuclear stain. Magenta arrowheads: putative pyramidal neurons. White arrowheads: putative endothelial cells. Scale =20μm B) Representative image of lateral ventricle 24 hours after intraventricular injection of 50ng IFNβ. IFN-brite (green), ISG IFITM3 (red), IBA1 (magenta) and DAPI (blue) with an inset (b) of IFN-brite cells lining the ventricle. Scale= 50μm. C) Representative image of striatum 24 hours after intraventricular injection of 50ng IFNβ. IFN-brite (green), NeuN (magenta), IBA1 (orange), and DAPI (blue). Inset (c) of IFN-brite (top) and merge with NeuN (bottom). White arrows indicate IFN responsive neurons. Scale= 10μm D) Model of induced developmental circuit remodeling: partial whisker deprivation in neonatal mice promotes circuit remodeling in the corresponding contralateral somatosensory cortex and leads to the emergence of IFN-responsive microglia (adapted from Escoubas et al 2024) E) Low power images of layer 5 control and whisker deprived barrel cortex. Inset of IFN-responsive microglia in Layer 6 deprived cortex. IFN-brite (green), IFITM3 (magenta), IBA1 (red), and DAPI (blue). Yellow arrowheads: IFN-brite+IFITM3+ microglia. scale= 40μm (left) and 10μm (inset).

We next examined IFN-brite expression in the brain, where IFN-I has functional impacts on brain development and homeostasis(9) via tonic signaling. We first examined which CNS cell types were competent to express IFN-brite by examining high power images of the tissues in Fig 2B, focusing on layer 5 of somatosensory cortex. In vehicle treated conditions, some endothelial cells were detected in vehicle treated conditions, consistent with our flow cytometry data (FIg 2C), but other cell types were rarely detected. After 24 hours of IFNβ exposure, multiple cell types expressed high levels of IFN-brite, including putative neurons, endothelial cells, and other cell types, some expression was detected (Fig. 3A). Ependymal cells lining the lateral ventricle were particularly strong expressors of IFN-brite after IFNβ exposure (Fig. 3B), as were subsets of neurons in the striatum (Fig. 3C), which is composed primarily of inhibitory medium spiny cells.

We previously adapted a model of experience-dependent circuit remodeling that induces a distinct population of IFN-responsive microglia in layer 5 of somatosensory cortex(9). The model involves partially lesioning mouse whiskers in early life, which induces rearrangement of the corresponding somatosensory cortex to match the new topographic map (Fig. 3C). As there are three synaptic connections between the afferent sensory whisker and the brain, this paradigm induces remodeling, but not inflammation. We previously showed that this sterile remodeling paradigm leads to expansion of IFN-responsive microglia using single cell sequencing and immunostaining for the ISG IFITM3 (9). To test whether IFN-brite could detect these remodeling-associated IFN-responsive microglia we performed the whisker-deprivation paradigm and co-labeled with our antibody-based methods for detecting these cells via expression of IFITM3 (Fig. 3D). We observed an expansion of IFN-brite+ microglia, most of which were also positive for IFITM3 protein. This supports our previous findings and suggested that IFN responses could be detected in response to physiologic perturbations that induce circuit remodeling in the developing CNS.

To determine whether IFN-brite affects the expression of native ISGs, we isolated microglia in naive postnatal day 10 brains using Magnetic-Activated Cell sorting (MACs), and performed quantitative PCR for a variety of ISGs. (Fig. S2A). We found that baseline levels of *Isg15* and other ISGs (*Bst2, Ifitm3, Ifi204*, etc) were largely unchanged (Fig. S2B). This suggests that the reporter is suitable for detecting IFN-responses and does not affect baseline IFN responsiveness. It is worth noting that globally *Isg15* deficient mice have impaired antiviral responses in response to some pathogens including influenza A (18-20). In summary, we expect this reporter to be suitable for most studies examining IFN signaling, but it would be prudent to test *Isg15* levels in the setting of specific experimental perturbations of interest.

### IFN-brite detects IFN responses in lung stromal and hematopoietic cells after Influenza A infection

**Figure 4:**
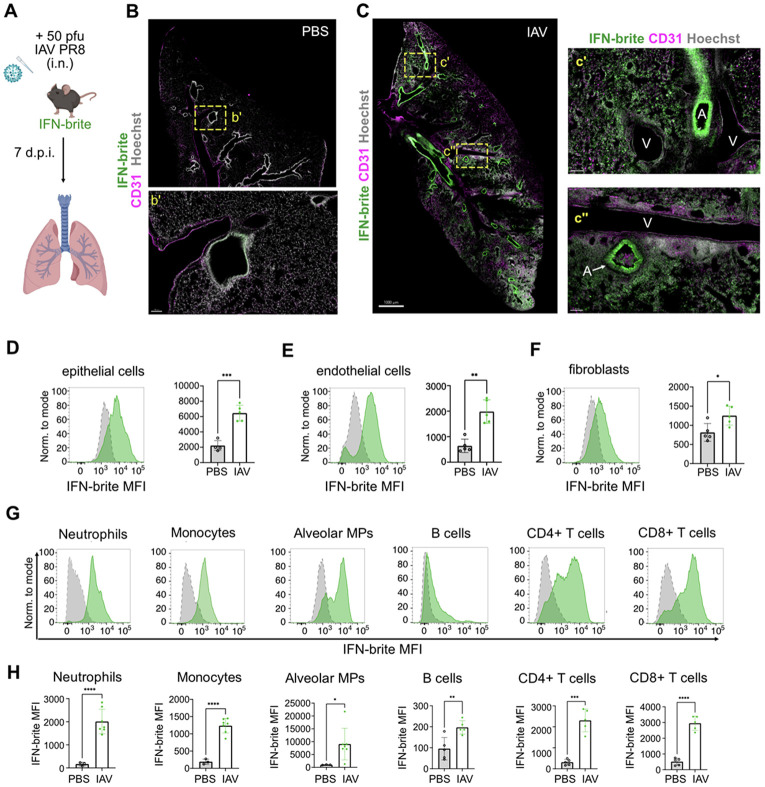
IFN-brite detects IFN responses in lung stromal and hematopoietic cells after Influenza A infection. A) Schematic of influenza A infection paradigm B) Representative low power image of non-infected lung (PBS vehicle only) in IFN-brite animals (scale=1mm); inset shows rare IFN-brite+ signal at an airway (scale=100μm). C) Representative low power image and insets of IAV PR8 infected lung 7 days post infection (DPI). Scale=1mm. Insets show IFN-brite+ airways (A) and parenchyma. V=vessels. D) Histogram and quantification of IFN-brite intensity (MFI) in epithelial cells in vehicle and IAV infected lungs. Data points represent mice from 2 independent experiments; unpaired t-test with Welch’s correction. E) Histogram and quantification of IFN-brite intensity (MFI) in endothelial cells in vehicle and IAV infected lung F) Histogram and quantification of IFN-brite intensity (MFI) in fibroblasts in vehicle and IAV infected lung G) Representative histograms of IFN-brite expression in vehicle (gray) and IAV infected (green) lung immune cell subsets. See Fig. S4 for gating strategy. H) Flow cytometric quantification of IFN-brite in lung immune cell subsets, data points represent mice from 2 independent experiments; unpaired t-test with Welch’s correction.

Although IFNs are critical for antiviral responses, including after lung influenza infection, their role and kinetics during influenza A viral (IAV) infection are less understood, due to the dual functions of Type I and Type III IFNs and variation in mouse and viral strains(3). Therefore, we next examined whether IFN-brite could be used to characterize IFN-responsive cell types during IAV infection. We infected adult IFN-brite mice with sublethal doses of Influenza A/PR/8/34 (H1N1) (IAV PR8; 50 pfu) (Fig. 4A), and collected lungs at 7 days post-infection (dpi) for imaging and flow cytometry. Infected IFN-brite mice did not display significant differences in sickness severity or weight loss compared to infected non-reporter counterparts (Fig. S4A).

We observed minimal IFN-brite expression in vehicle-treated lungs and robust IFN-brite induction after IAV exposure, including airway epithelial cells and throughout the lung parenchyma (Fig. 4B-C). We quantified IFN-brite induction using flow cytometry, dissociating via enzymatic digestion to collect both immune and stromal subsets (see methods). We observed robust induction of IFN-brite after IAV infection in epithelial and endothelial cells, and to a lesser extent in fibroblasts at this timepoint (Fig. 4D-F; gating strategy in Fig. S4). These data suggest that IFN-brite reliably detects IFN responses across multiple stromal cell subsets.

Consistent with prior reports (10), we also found significantly increased expression of IFN-brite in lung hematopoietic cells, including neutrophils, Ly6C^hi^ monocytes, alveolar macrophages, CD8+ T cells, and CD4+ T cells (Fig. 4G-H). We did not observe significant increases in ISG15-mGL in splenic hematopoietic cells, except in circulating neutrophils and Ly6C^hi^ monocytes (not shown), consistent with a prior report that IFN response to IAV is primarily confined to the lung and draining lymph nodes(10). Overall, these data reveal that IFN-brite can detect IFN responses across cell types, organs, and perturbations.

## Discussion

This study reports a broadly useful new tool for studying IFN responses in both physiologic and immune activated settings. The detection of robust IFN-responses in multiple CNS cell types, including neurons, astrocytes, and ependymal cells reveals that many cell types are competent to respond to IFN-signaling. While this is not surprising, as all CNS cell types express high levels of *Ifnar1* (9), it raises the question of how IFN signaling shapes cell function beyond the hematopoietic system. Further variations of this reporter incorporating a nuclear localization signal would greatly facilitate single nuclear sequencing of IFN-responsive neurons for further functional characterization.

In addition, our data shows that IFN-brite can be used to track virally-induced IFN responses in the lung. Our data show that both hematopoietic and non-hematopoietic cell types IFN responses, which differs from a prior reporter which was detected predominantly in hematopoietic cells after influenza infection(10). This may be partly due to the increased sensitivity of our reporter. For example, it detects the majority of IFN-responsive microglia after whisker deprivation (Fig. 3), whereas Mx1^GFP^ detected only 25% of IFN-responsive microglia in the same paradigm (9). However, it is also possible that *Isg15* reports on a broader array of cell types than *Mx1,* or has slightly different patterns of IFN-induced expression.

IFN signaling is dysregulated in a variety of neurodevelopmental disorders (NDDs) including in Type I interferonopathies (1, 21, 22). While the classic interferonopathies are rare but involve extremely high level IFN signaling, many more neurodevelopmental disorders include lower level but significant amounts of IFN dysregulation. For example, Down syndrome is one of the most prevalent genetic neurodevelopmental disorders and is considered a ‘mild interferonopathy’ due to the presence of multiple IFN genes on triplicated chromosome 21 and consequent IFN dysregulation (23-26). Early life viral infection is a strong risk factor for later neurodevelopmental and psychiatric diagnoses (27, 28), suggesting the importance of better defining IFN-responsive cells in animal models of these disorders. As such, this reporter may yield a better understanding of IFN signaling in the brain and across tissues, which may have therapeutic implications (29) including for neurodevelopmental disorders.

## Brief methods

### Mice

All mouse strains were maintained in the University of California, San Francisco specific pathogen–free animal facility, and all animal protocols were approved by and in accordance with the guidelines established by the Institutional Animal Care and Use Committee and Laboratory Animal Resource Center. Littermate controls were used for all experiments when feasible, and all mice were backcrossed > 10 generations on a C57BL/6 background unless otherwise indicated. IFN-brite animals were backcrossed for 3 generations prior to experiments to breed out potential indels. All experiments were performed in heterozygous animals and incorporated animals of both sexes in approximately equal numbers.

### Isolation and culture of primary glia cells

Mixed glia cultures were generated from P2-P4 pups from wild-type or IFN-brite heterozygous backgrounds and grown in T75 flasks with DMEM supplemented with heat inactivated 10% FBS and 1% Pen/Strep at 37°C 5% CO2. The media was changed the next day and cultures were grown for another 10-12 days. Microglia were detached by hitting the flasks 10x against the bench, stained with Trypan blue and counted using a Countess 3 Automated Cell Counter (Invitrogen). Then cells were plated in a 96-well plate at a density of 30,000 cells/well in 200ul final volume (3-6 replicate wells per condition). The next day, microglia were treated with vehicle or recombinant cytokine at the indicated dosages, then fixed in 4% paraformaldehyde after either 16 or 24 hours incubation and immunostained.

### *Tissue preparation for immunostaining and* in situ *hybridization*

For all immunohistochemistry and *in situ* hybridization experiments mice older than P7 and mice P7 or younger were anesthetized and perfused transcardially with PBS followed by 4% paraformaldehyde (PFA) fixative. Tissues were post-fixed overnight and then transferred to a 30% sucrose solution for a minimum of 2 days. Tissues were then embedded in OCT (VWR 25608-930), frozen on dry ice, and stored at −80°C until sectioning. For immunohistochemistry experiments, floating brain sections of 40 μm or 50 μm thickness or lung sections of 200 μm thickness were cut using a Cryostar NX70 cryostat (Thermo).

### Immunohistochemistry, confocal microscopy, and image acquisition

For immunohistochemistry, floating brain sections (40 μm, 50μm, or 300 μm in thickness) were incubated in the Staining Solution (0.4% TritonX-100, 5% normal goat serum, 1X PBS) for one hour. Primary antibodies were diluted in Staining Solution, and tissue was incubated in primary solution on a shaker overnight at 4°C. Secondary antibodies were diluted in Staining Solution, and tissue was incubated on a shaker for 2 hours at room temperature. DAPI (Thermo) was added at 1:1000 during the last 5 minutes of incubation. Brain sections were mounted on slides and sealed onto coverslips with ProLong Glass without DAPI (Thermo) for high-resolution imaging, SlowFade Glass (Thermo) for 300 μm section immunostaining, and Fluoromount-G (SouthernBiotech) for all other experiments.

Slides were imaged on an LSM 700 confocal microscope (Zeiss) was used with 20x, 40x, and 63x objectives. Within experiments, laser powers and gain were consistent.

For lung immunohistochemistry, floating lung sections (200 μm in thickness) were washed and incubated in permeabilization buffer (DPBS/0.2%Triton X-100/0.3M glycine) for 4-5 hours at room temperature, then blocked in DPBS/0.2% TritonX-100/5% serum (from the same host species as the secondary antibody) at room temperature overnight. After, samples were washed in DPBS/0.2% Tween-20 once and incubated with primary antibodies diluted in DPBS/0.2% Tween-20/5% serum, room temperature until the next day. Next, samples were washed in DPBS/0.2% Tween-20 for 30 min, 3–4 times, then incubated with secondary antibodies diluted in DPBS/0.2%Tween-20/5% serum at room temperature overnight. Samples were washed in PBS for 2hrs, then cleared by incubating in refractive index matching solution (RIMS; 80% Histodenz in 1X PBS, 0.01% sodium azide, 0.1% Tween20) until transparent, and then mounted in fresh RIMS solution. Slides were imaged on a Leica DMi8 Stellaris 8 Tau STED-FLIM Confocal Microscope with HC PL APO 10x/0.4 NA CS2 and HC PL APO 20x/0.75 NA CS2 objectives. Laser powers and gain were consistent within experiments.

### Stereotaxic injections

All brain injections were performed with a Kopf stereotaxic apparatus (David Kopf, Tujunga, CA) and a microdispensing pump (World Precision Instruments) holding a Hamilton Syringe (model 701 RN, 10 ml) with a beveled glass needle (~50 mm outer diameter). For intraventricular injections into neonates (P0-2), mice were anesthetized by hypothermia on ice for 3 min. Bilateral i.c.v. injection of cytokine was performed (from lambda: 1.5 mm AP, +/− 0.8 mm ML, −1.75 mm DV). Afterwards, pups were warmed on a heating pad until full recovery before returning to their home cage.

### Brain dissociation for flow cytometry

P4-5 mice were deeply anesthetized via hypothermia and transcardially perfused with 5-10 mL of ice cold 1X PBS via the left ventricle. Brains were isolated, hemi-sected, and collected into 2 mL of iMED+ (isolation medium: 1X HBSS (without calcium or magnesium) supplemented with 10 mg/mL phenol red, 15 mM HEPES, and 0.6% glucose). Hemibrain samples were then homogenized using a “cold protease” protocol optimized to isolate astrocytes, endothelial cells, and hematopoietic cells (including microglia), adapted from (17). Samples were digested on ice for 20-25 minutes in 1X DPBS (no calcium or magnesium) supplemented with 5 mg/mL subtilisin A (Protease from *Bacillus licheniformus,* Sigma #P5380) and 20 μg/mL DNase I (Roche #10104159001) with repeated trituration using a P1000 pipette every 5 minutes. Once homogenized, digestion was halted using a solution containing protease inhibitors (“LO-OVO”: 1X HBSS without calcium/magnesium supplemented with 10 mg/mL phenol red, 0.08% glucose, 1 mg/mL ovomucoid (Worthington), 1 mg/mL bovine serum albumin (Sigma), 20 μg/mL DNAse I). Digested samples were filtered through a 70 μm cell strainer and cells were collected via centrifugation at 300xg for 10 minutes at 4°C. Cell pellets were then resuspended in a 22% Percoll solution (GE Healthcare) with a 1X DPBS layer floated on top, as previously described (30). Samples were centrifuged at 900xg for 20 min (acceleration=4, brake=0) at 4°C and the supernatant layers were discarded to remove myelin and debris. Cell pellets were resuspended prior to antibody staining for flow cytometry.

### Lung dissociation for flow cytometry

Single cell suspensions were prepared from lung and spleen tissues as described previously(31-33). Briefly, mice were euthanized with CO_2_. Immediately after, mice were perfused by flushing the left ventricle with 10 mL 1X DPBS, and whole lungs (left lobe) were excised. Tissues were minced into small pieces (<1 mm). For flow cytometry of immune panels, samples were digested in 1X Hanks’ Balanced Salt Solution (HBSS) with 0.2 mg/mL Liberase Tm (Roche, Cat# 5401127001) and 25 μg/mL DNase 1 (Roche, Cat# 10104159001) for 30 min at 37°C on a shaker. For flow cytometry of stromal cells, samples were digested in PBS with 7.5 U/mL Dispase II (Gibco, ThermoFisher Cat# 17105041), 112 U/mL Collagenase I (Gibco, ThermoFisher Cat# 17100017), and 40 μg/mL DNase 1 (Roche, Cat# 10104159001) for 30 min at 37°C on a shaker. Tissue pieces were then sheared through a 18g needle, followed by filtration through 70μm filters, washed, and subjected to red blood cell lysis (1X Pharm-Lyse lysing solution; BD Biosciences) before final suspension in FACS buffer (1X DPBS, 3% FBS, 0.05% NaN_3_).

### Staining for flow cytometry

For brain samples, single cell suspensions were stained in iMED- (1X HBSS without calcium/magnesium/phenol red supplemented with 15 mM HEPES and 0.6% glucose) containing fluorophore-conjugated antibodies, viability dye (Draq7, 1:1000, Biolegend), and Fc Block (2.4G2, 1:100, BD Biosciences) for 45 min at 4°C. Cells were washed with iMED-, pelleted at 300xg for 5 min at 4°C, and resuspended in iMED- prior to analysis. Data was collected on a FACSAria Fusion (BD).

For lung samples, single cell suspensions were stained in FACS buffer (1X DPBS, 3% FBS, 0.05% NaN_3_) containing fluorophore-conjugated antibodies, viability dye (Draq7, 1:1000, Biolegend), and Fc Block (2.4G2, 1:100, BD Biosciences) for 45 min at 4°C. Cells were washed with FACS buffer, pelleted at 1200rpm for 2 min at 4°C, and resuspended in FACS buffer prior to analysis. Data was collected on a dual Fortessa X20 (BD) or FACSAria Fusion (BD). Analysis of flow cytometry data was performed using FlowJo v10 software (BD).

### Influenza A infection

Mice were anesthetized with isoflurane. Influenza A/PR/8/34 (H1N1) (ATCC, VR-95) was administered intranasally through each nostril (for a total of 40 μl volume, 50 pfu) using a P200 pipette. After administration, mice were held with nostrils upright for 10–15 s to ensure complete inhalation of the virus. Infected mice were monitored daily from the day of infection until the specified time points for tissue collection.

### Quantification and statistical analyses

Graphpad Prism 9.4.1 was used for most statistical analyses. Statistical tests are as described in the text and figure legends.

### Antibodies for flow cytometry:

**Table:** 

Antigen– Fluorophore	Clone	Dilution	Vendor	Catalog #
CD11b–Pacific Blue	M1/70	1:200	Biolegend	101224
CD45–BUV395	30-F11	1:400	BD Biosciences	564279
ASCA-2–APC	IH3-18A3	1:100	Milltenyi Biotec	130-117-386
CD31–PE-vio770	390	1:100	Milltenyi Biotec	130-102-375
CD206–BV605	C068C2	1:200	Biolegend	141721
CD3–PE	17A2	1:200	Biolegend	100206
CD31–BV421	390	1:400	Biolegend	102424
Sca-1–BV711	D7	1:200	Biolegend	108131
CD8α–BV785	53-6.7	1:200	Biolegend	100750
PDGFRα–APC	APA5	1:200	Biolegend	135908
EpCAM–APC-Fire750	G8.8	1:200	Biolegend	118230
CD4–BV711	RM4-5	1:200	Biolegend	100557
TCRγδ–PerCP-Cy5.5	GL3	1:200	Biolegend	118118
Ly-6C–BV605	HK1.4	1:400	Biolegend	128035
NK1.1–BV650	PK136	1:200	Biolegend	108736
CD64–AF647	X54-5/7.1	1:200	BD Biosciences	558539
Ly-6G–APC-Cy7	1A8	1:200	Biolegend	127624
MHCII–BUV737	M5/114.15.2	1:400	BD Biosciences	748845
CD19–PE-Dazzle594	6D5	1:400	Biolegend	115554
CD11c–PE-Cy7	N418	1:200	Biolegend	117318
Siglec-F–BV786	E50-2440	1:200	BD Biosciences	740956

### Antibodies for immunofluorescence:

**Table:** 

Antigen	Clone	Dilution	Vendor	Catalog #
Chicken anti-GFP	polyclonal	1:5000	Aves	GFP-1020
Guinea Pig anti-GFP	polyclonal	1:1000	Synaptic Systems	132 004
Goat anti-GFP	polyclonal	1:500	Abcam	ab6673
Rat anti-CD31	MEC 13.3	1:500	BD	553370
Rabbit anti-IFITM3	polyclonal	1:500	Proteintech	11714-1-AP
Guinea Pig anti-IBA1	Gp311H9	1:2000	Synaptic Systems	234 308
Rabbit anti-IBA1	polyclonal	1:1000	Wako	019-19741
Chicken anti-NeuN	polyclonal	1:500	Millipore	ABN91
Rat anti-BST2	44E9R	1:500	R&D Systems	MAB8660-SP

## Supplementary Material

Supplement 1
