# Supplementary material for: A genetically encoded reporter reveals interferon responses in multiple cell lineages
Source: bioRxiv. 2026 Jan 10:2026.01.08.698513. Preprint. [Version 2] doi: 10.64898/2026.01.08.698513 (PMC12803042; doi:10.64898/2026.01.08.698513)
Supplement: Supplement 1 [file NIHPP2026.01.08.698513v2-supplement-1.pdf]

bioRxiv preprint doi: https://doi.org/10.64898/2026.01.08.698513; this version posted January 10, 2026. The copyright holder for this preprint (which was not certified by peer review) is the author/funder, who has granted bioRxiv a license to display the preprint in perpetuity. It is made available under aCC-BY 4.0 International license.

Supplementary Figures

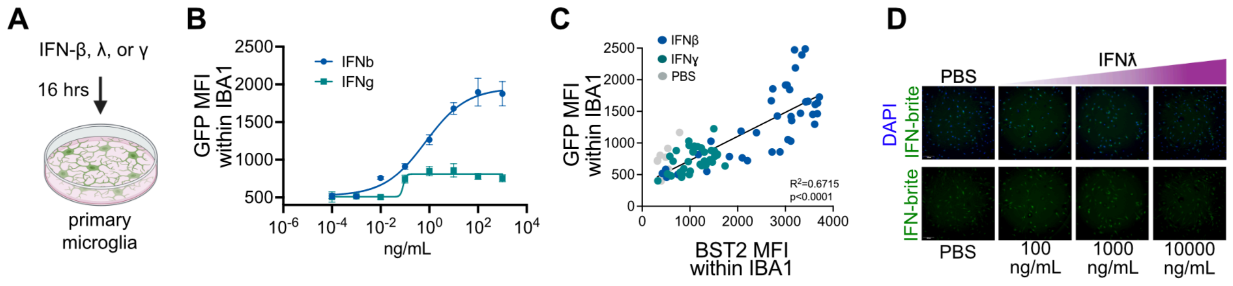

Figure S1: In vitro microglial responses to 16 hour time window of IFN administration.

A) Design of in vitro assay using mouse primary microglia to detect IFN responses. Indicated cytokines were added for 16 hours prior to fixation, antibody staining, and imaging.

B) Best-fit curve of IFN-brite expression (anti-GFP) in microglia after 16 hours of varying doses of IFN-β and IFN-γ by mean fluorescence intensity (MFI) within masked IBA1 signal. n=6 well, 2 independent experiments per group.

C) Correlation of IFN-brite expression (GFP) in microglia (by MFI) with protein staining of the ISG, BST2.

D) Representative images of in vitro microglial responses to varying doses of IFNλ2. DAPI (blue) IFN-brite (green).

bioRxiv preprint doi: https://doi.org/10.64898/2026.01.08.698513; this version posted January 10, 2026. The copyright holder for this preprint (which was not certified by peer review) is the author/funder, who has granted bioRxiv a license to display the preprint in perpetuity. It is made available under aCC-BY 4.0 International license.

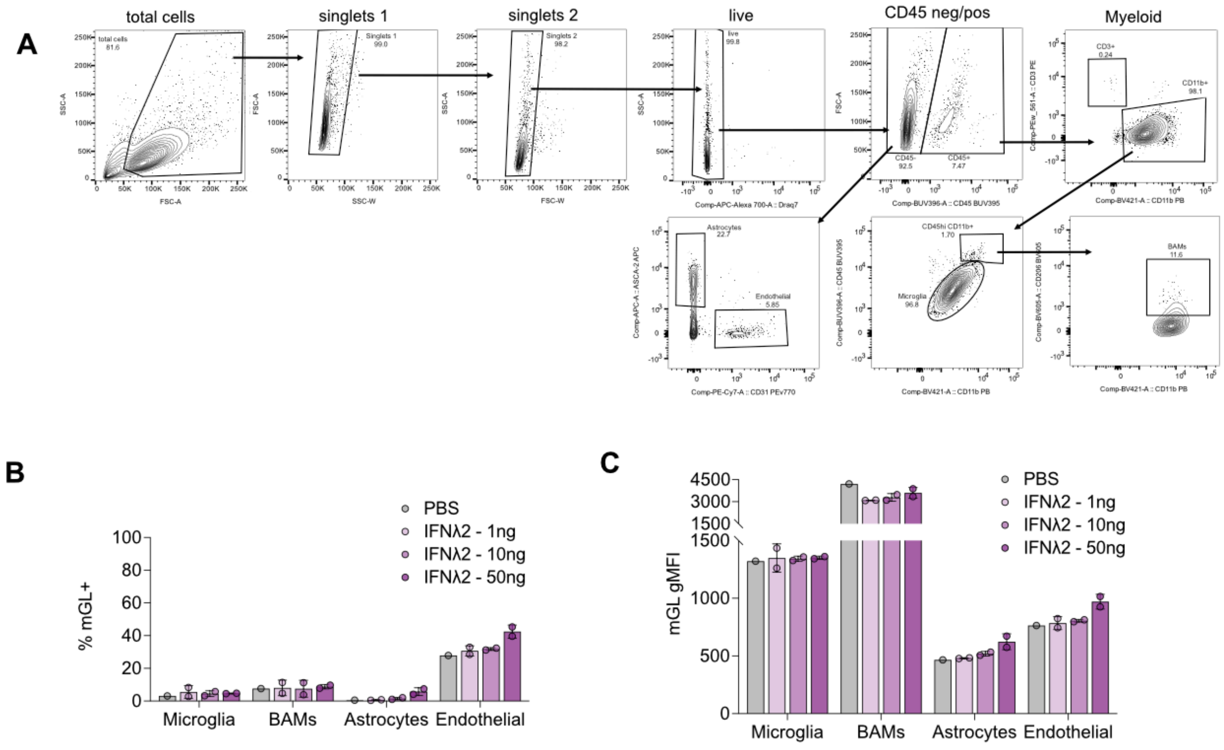

Figure S2: Gating strategy for sorting of CNS cells and additional data on intracerebroventricular injection of IFNλ2, related to Figure 2.

A) Gating strategy for flow cytometry of myeloid cells, astrocytes, and endothelial cells.
B) Percent of cell types responding to varying doses of IFNλ2. n=1 PBS, n=2 1, 10, and 50ng doses.
C) IFN-brite expression by geometric MFI (gMFI) in response to varying doses of IFNλ2. n=1 PBS, n=2 1, 10, and 50ng doses.

bioRxiv preprint doi: https://doi.org/10.64898/2026.01.08.698513; this version posted January 10, 2026. The copyright holder for this preprint (which was not certified by peer review) is the author/funder, who has granted bioRxiv a license to display the preprint in perpetuity. It is made available under aCC-BY 4.0 International license.

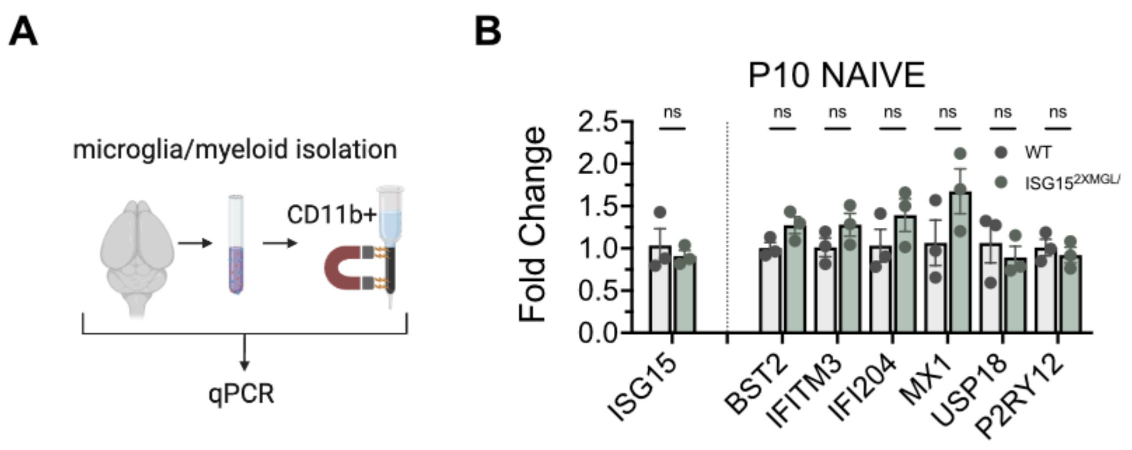

Figure S3: Native ISG expression in IFN-brite mice compared to wild type controls, related to Figure 3.

A) Microglia/myeloid magnetic bead isolation strategy
B) Fold change relative to wildtype littermates of indicated ISGs as well as microglial marker *P2ry12* in naive P10 brain myeloid cells by quantitative reverse transcriptase PCR (normalized to housekeeping gene, Rps17), n=3 per group.

bioRxiv preprint doi: https://doi.org/10.64898/2026.01.08.698513; this version posted January 10, 2026. The copyright holder for this preprint (which was not certified by peer review) is the author/funder, who has granted bioRxiv a license to display the preprint in perpetuity. It is made available under aCC-BY 4.0 International license.

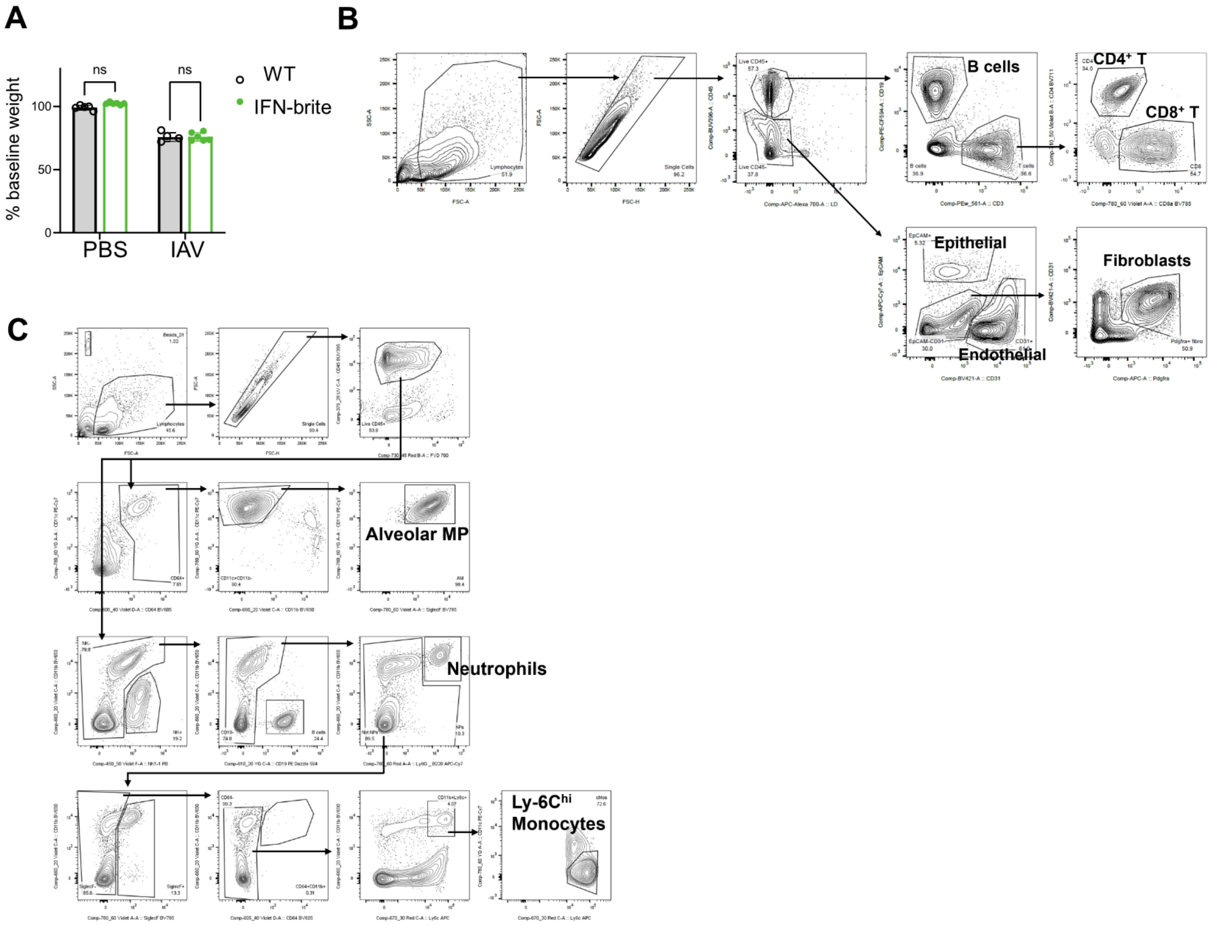

Figure S4: Influenza A infection severity in IFN-brite mice, and gating strategy for lung flow cytometry, related to Fig. 4

A) Body weight (as percent of baseline day 0 weights) of PBS-treated versus IAV-infected WT and IFN-brite mice at 8 dpi. n=5-6 per group, 2 independent experiments, two-way ANOVA with Sidak's multiple comparisons test.
B) Gating strategy for stromal and lymphoid cells analyzed in Fig. 4.
C) Gating strategy for myeloid cells analyzed in Fig. 4. (MP = macrophages)
